# Effect of N-acetylcysteine on exacerbations of bronchiectasis (BENE): a randomized controlled trial

**DOI:** 10.1186/s12931-019-1042-x

**Published:** 2019-04-11

**Authors:** Qian Qi, Yirepanjaing Ailiyaer, Ruijuan Liu, Yan Zhang, Caiyu Li, Mingtao Liu, Xiuxiu Wang, Lijun Jing, Yu Li

**Affiliations:** 1 Department of Respiratory Medicine, Qilu Hospital of Shandong University, No. 107 Wenhua Xilu, Jinan, 250012 Shandong Province China; 2 Department of Respiratory Medicine, Jinan City People’s Hospital, Jinan, China; 3 Department of Respiratory Medicine, The Fifth Affiliated Hospital of Xinjiang Medical University, Xinjiang, China; 4 Department of Respiratory Medicine, Jining No.1 People’s Hospital, Jining, China; 5 Department of Respiratory Medicine, The Fourth People’s Hospital of Jinan, Jinan, China; 6 The Second Hospital of Shandong University, Jinan, China; 7 Department of Respiratory Medicine, Binzhou People’s Hospital, Binzhou, China

**Keywords:** N-acetylcysteine, Bronchiectasis, Long-term therapy, Exacerbations

## Abstract

**Background:**

N-acetylcysteine is a classic mucolytic agent. This study aimed to investigate the efficacy of N-acetylcysteine on reducing the risk of exacerbations in bronchiectasis patients.

**Methods:**

A prospective, randomized, controlled trial was conducted between April 1, 2014 and December 31, 2016 in five general hospitals in Shandong Province, China. Adult bronchiectasis patients with at least two exacerbations in the past year were potentially eligible. Patients were randomly assigned to receive oral N-acetylcysteine (600 mg, twice daily, 12 months) or on-demand treatment.

**Results:**

A total of 161 patients were eligible for randomization (81 to the N-acetylcysteine group and 80 to the control group). During the 12-month follow-up, the incidence of exacerbations in the N-acetylcysteine group was significantly lower than that in the control group (1.31 vs. 1.98 exacerbations per patient-year; risk ratio, 0.41; 95% CI, 0.17–0.66; *P* = 0.0011). The median number of exacerbations in the N-acetylcysteine group was 1 (0.5–2), compared with 2 (1–2) in the control group (U = − 2.95, *P* = 0.003). A total of 24.7% of the N-acetylcysteine group patients and 11.3% of the control group patients remained exacerbation-free throughout the 12-month follow-up (*χ*^*2*^ = 4.924, *P* = 0.026). Compared with the control group, the volume of 24-h sputum in the N-acetylcysteine group was significantly reduced (*t* = − 3.091, *P* = 0.002). Additionally, the N-acetylcysteine group showed a significant improvement in the quality of life. No severe adverse events were reported in the intervention group.

**Conclusion:**

The long-term use of N-acetylcysteine is able to reduce the risk of exacerbations for bronchiectasis patients in Shandong Province, China. The results of this study should be verified in a larger randomized controlled trial.

**Trial registration:**

ClinicalTrials.gov (NCT02088216) (Registered date: March 5, 2014).

## Introduction

Bronchiectasis is a chronic suppurative lung disease characterized by permanent dilation of bronchi and bronchioles [1]. The number of hospital admissions due to bronchiectasis are increasing [2]. Pathophysiologically, the hypertrophy of mucus-secreting glands leads to excessive secretion of mucus into the airway. The normal clearance of the mucociliary system is compromised in patients with bronchiectasis, resulting in pooling of mucus within the airway [3]. Excessive secretion and retention of mucus in the airway results in a chronic cough and continuous expectoration in patients with bronchiectasis. Inflammatory reactions, injury and distortion of bronchi, mucus retention, and respiratory infection or bacterial colonization are the four major components of the “vicious cycle” underlying the pathogenesis of bronchiectasis [4, 5]. The population of patients with bronchiectasis is extremely heterogeneous. Aliberti et al. [6] reported that “dry bronchiectasis” is an established clinical phenotype (27% of patients), and none of these patients had chronic infections or daily sputum production. The other three clusters of bronchiectasis were characterized by the presence of chronic infection with *Pseudomonas aeruginosa* or other pathogens and daily sputum production [6]. Mucus production plays an essential role in the other three clusters of bronchiectasis. It has been deduced that reducing the production of mucus or improving the clearance of sputum in the airway is the key to break down this “vicious cycle” and enhance the therapeutic efficacy for bronchiectasis.

Mucoactive drugs are commonly used to clear the airway in mucus hypersecretion diseases [7]. Mucoactive agents, such as hypertonic saline, mannitol, and erdosteine, have greatly improved the treatment outcomes for bronchiectasis. It was found that a short-term regimen of erdosteine plus routine chest physiotherapy decreased mucus hypersecretion in stable patients with bronchiectasis [8]. Another clinical trial showed that the inhalation of dry powder mannitol for 12 weeks significantly reduced the amount of sputum in bronchiectasis patients [9]. Unfortunately, these trials were limited by their small sample sizes and short treatment period. A systematic review on the efficacy of mucolytics for bronchiectasis indicated that the level of evidence supporting the routine use of mucoactive agents for bronchiectasis was low [10]. Therefore, large randomized controlled trials are needed to generate high level evidence on the efficacy of the long-term use of mucolytics for reducing the risk of exacerbations in patients with bronchiectasis.

Currently, investigators have found that N-acetylcysteine, an effective mucolytic agent, not only reduces the viscosity and elasticity of sputum, but it also has anti-inflammatory and antioxidant activity [11, 12]. Moreover, N-acetylcysteine has shown a protective effect against the detrimental impact of lipopolysaccharide in vitro [13]. In addition, a randomized, placebo-controlled trial with a large sample size demonstrated that a high dose of oral N-acetylcysteine (1200 mg/day) for 1 year significantly reduced the rate of exacerbations and improved the quality of life in patients with chronic obstructive pulmonary disease (COPD) [14, 15]. Additionally, the Spanish guidelines on the treatment of bronchiectasis indicate that the use of N-acetylcysteine should be considered for patients with bronchiectasis and COPD [16]. Furthermore, Oliveria et al. found that the oxidative stress biomarkers of adult bronchiectasis patients were significantly elevated in both cellular and plasma extracts [17], suggesting that oxidative stress plays an important role in the pathophysiological changes of bronchiectasis. Therefore, as a classic mucolytic agent with antioxidant and anti-inflammatory properties, N-acetylcysteine can be effective in the treatment of bronchiectasis.

In this study, we aimed to assess whether the long-term use of oral N-acetylcysteine (600 mg, twice daily) might reduce the rate of exacerbations and improve the quality of life in patients with bronchiectasis.

## Materials and methods

### Study subjects

Bronchiectasis was diagnosed based on the clinical manifestations and imaging features of high-resolution computed tomography scans (HRCT), including the internal lumen diameter of the bronchi being greater than that of the accompanying pulmonary artery, the bronchi failing to taper in the periphery of the chest, or the bronchi terminating in a cyst [5]. The underlying etiology of bronchiectasis was determined after performing a panel of investigations according to the 2010 British Thoracic Society guidelines for bronchiectasis and our previous studies on the etiology of bronchiectasis [5, 18]. Patients were screened for potential eligibility in five general hospitals in Shandong Province, China, from April 1, 2014 to December 31, 2016. The inclusion criteria were as follows: 1) subjects aged between 18 and 80 years old; 2) a diagnosis of idiopathic or post-infective bronchiectasis was made according to Qi et al. [18]; and 3) patients had at least two exacerbations in the past year and were in a stable state for at least 4 weeks prior to randomization. Patients were excluded if they fulfilled any of the following criteria: current smokers; cigarette smoking within 6 months; cystic fibrosis or other etiologies (such as immunodeficiency, allergic bronchopulmonary aspergillosis, traction bronchiectasis caused by emphysema, advanced pulmonary fibrosis, etc.); pulmonary function test results showing a forced expiratory volume in 1 s (FEV_1_) ≤ 30% of the predicted value; primary diagnosis of COPD or asthma; a history of severe cardiovascular disease; comorbidity with liver disease, kidney disease, malignant tumor, gastric ulcer, or intestinal malabsorption; a known allergy to N-acetylcysteine; pregnancy or lactation (for women); a history of prior macrolide use of more than 1 week; and poor compliance. This study was registered at *ClinicalTrials.gov* (NCT02088216) and was approved by the ethics committees of the five participating hospitals. Informed consents were obtained from all patients.

### Study design

The Effect of N-acetylcysteine on Exacerbations of Bronchiectasis (BENE) study was a prospective, randomized, controlled, multicenter clinical trial. The main study objective was to assess whether the long-term use of oral N-acetylcysteine (600 mg, twice daily, 12 months) might reduce the incidence of exacerbations and improve the quality of life in patients with bronchiectasis. According to previous clinical data on exacerbations of bronchiectasis [18] and the inclusion criteria of this study that a subpopulation of bronchiectasis patients with at least two exacerbations in the past year were included, we hypothesized that the baseline frequency of exacerbations was 3. Due to the lack of clinical data on the efficiency of N-acetylcysteine in the treatment of bronchiectasis, we estimated that approximately 154 patients would need to be enrolled in this study to have 80% power to detect a reduction of at least 33% in the yearly exacerbation incidence, assuming a two-sided α level of 0.05 and 20% attrition.

### Methods

The baseline data of the enrolled patients were analyzed. The disease severity of bronchiectasis was evaluated according to a validated severity classification system for bronchiectasis—the Bronchiectasis Severity Index (BSI) [19]. The sequence was generated by a computer, and the patients who met the criteria were allocated in a 1:1 ratio to receive either oral N-acetylcysteine (600 mg, twice daily) or control (receive as-needed therapy) for 12 months. All the participants were followed for up to 12 months. N-acetylcysteine was provided for patients of the N-acetylcysteine group each month to potentially promote treatment adherence, and telephone calls from the study site were used to remind the patients of treatment and to check for treatment compliance. Outpatient and/or telephone visits were obtained for the primary and secondary outcomes at 1, 3, 6, 9, and 12 months. Each visit included assessment of respiratory signs and symptoms, a physical examination, adverse events, compliance, and exacerbations of bronchiectasis, etc. When patients suffered from exacerbations of bronchiectasis, the treatment strategy based on current guidelines was instituted [20]. Meanwhile, patients in the N-acetylcysteine group were also required to receive oral N-acetylcysteine (600 mg, twice daily) during the study period. For the control group, interventions would be discontinued once the patient recovered from the exacerbation. Maintenance therapies that had already started in patients with pulmonary ventilation dysfunction prior to enrollment, such as short-acting bronchodilators, long-acting bronchodilators, inhaled glucocorticoids, or theophylline, were continued during the study.

### Study end-points

The primary end-point was the incidence of exacerbations in a year, defined as the number of all exacerbations associated with bronchiectasis within 1 year. According to a consensus definition for clinical research, an exacerbation of bronchiectasis is defined as a bronchiectasis patient with a deterioration in three or more key symptoms (including cough, sputum volume and/or consistency, sputum purulence, breathlessness and/or exercise tolerance, fatigue and/or malaise, and hemoptysis) for at least 48 h and a clinician determining that a change in bronchiectasis treatment is required [21]. Details regarding symptoms of an exacerbation such as cough, sputum volume (collected over 24 h), sputum properties, breathlessness, fatigue and hemoptysis were documented by site investigators from all centers.

Secondary end-points included the percentage of patients remaining exacerbation-free throughout the 12-month follow-up, the time to first exacerbation, the time to second exacerbation, 24-h sputum volume, pulmonary function, inflammation indices, quality of life as assessed by the COPD assessment test (CAT) score [22], and adverse events. The CAT score is a valid and reproducible instrument in patients with bronchiectasis, presenting a good correlation with clinical, functional, and quality-of-life measurements [23]. It has been validated in a bronchiectasis patient population and was used to determine the quality of life of bronchiectasis patients in this study. The volume of 24-h sputum was measured by a graduated cylinder marked in milliliters. Eligible sputum samples were used for testing sputum microbiological culture and antibiotic resistance. Pulmonary function was assessed three times before recruitment, and the maximum value was used for analysis. Following treatment, pulmonary function was reassessed for forced vital capacity (FVC), FEV_1_, percentage of predicted FEV1, FEV_1_/FVC ratio (FEV_1_/FVC), and inspiratory capacity, etc. Inflammatory markers including serum C-reactive protein (CRP) and the erythrocyte sedimentation rate (ESR) were tested at each visit. The number of lobes involved was counted, and the degree of bronchiectasis was quantified by HRCT according to the modified Reiff grade [24]. Furthermore, the degree of dyspnea was assessed by the modified Medical Research Council (mMRC) scale [25].

### Statistical analyses

Patients who received at least one assessment visit after randomization were left in the original group for analysis of the results. Continuous data were expressed as the mean ± standard deviation and were compared using the Student’s *t* test. Categorical variables were expressed as a number (%) and were compared using the *χ*^*2*^ test. The number of exacerbations was described by the median (interquartile range, IQR). The Mann-Whitney U test was performed to compare the difference in the number of exacerbations between the two groups. The primary study end point was the incidence of exacerbations, which is a count and follows Poisson distribution. Negative binomial regression not including other covariates was employed to compute the risk ratio and 95% confidence intervals (CIs) for N-acetylcysteine vs. the control. The time to first exacerbation and the time to second exacerbation were described by Kaplan–Meier curves, and the log-rank test was used to explore the differences in the time to first exacerbation and the time to second exacerbation between the two groups. All analyses were conducted with the Statistical Package for the Social Sciences software (version 20.0). A *p* value < 0.05 was considered statistically significant.

## Results

### Patients

**Fig. 1:**
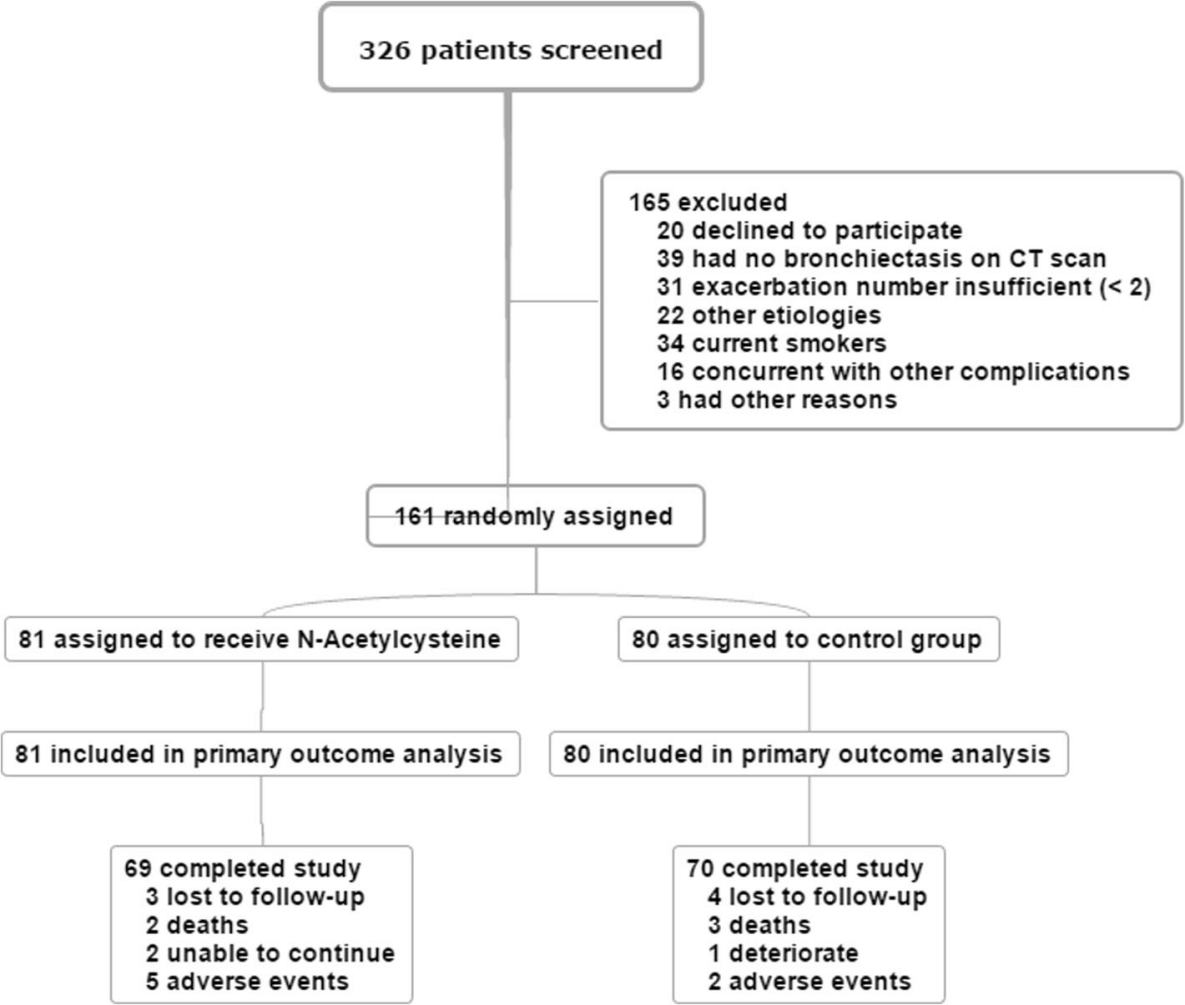
Flowchart of patient enrollment in this study

A total of 326 subjects were screened in this study, and 161 subjects underwent randomization from April 1, 2014 to December 31, 2016 (Fig. 1). Eighty-one of them received treatment with oral N-acetylcysteine, and 80 were in the control group. Twenty-two patients (13.7%) were excluded from the final analysis due to dropout, including 12 in the intervention group and 10 in the control group (14.8% vs. 12.5%; *χ*^*2*^ = 0.183, *P* = 0.669). The reasons for dropout are shown in Fig. 1. In the N-acetylcysteine group, one patient died of acute ischemic stroke and one patient died from acute exacerbation of bronchiectasis. In the control group, one patient died of coronary artery disease and two patients died from acute exacerbations of bronchiectasis. No deaths were considered by the investigators to be related to the study drugs. Finally, 69 patients (85.2%) in the N-acetylcysteine group and 70 patients (87.5%) in the control group completed the study. The baseline characteristics, including sex, age, body mass index, smoking history, etiology of bronchiectasis, HRCT grade, extent of the lesion lobes, mMRC score, CAT score, 24-h sputum volume, sputum culture of *P. aeruginosa* positive, number of exacerbations in the last year, inflammatory indices, lung function parameters, and BSI, were not significantly different between the intervention and control groups (Table 1). Additionally, baseline medications were not significantly different between the two groups (Table 1).

**Table 1:** Baseline characteristics of the study patients

Characteristic	Group		*P*-value
	Control group (*N* = 80)	N-acetylcysteine group (*N* = 81)	
Gender			
Female, n (%)	52 (65.0)	45 (55.6)	0.144
Age, years	56.56 ± 12.41	53.28 ± 11.90	0.089
Body mass index, kg/m^2^	22.16 ± 4.22	22.72 ± 3.57	0.362
Ex-smoker, n (%)	8 (10.0)	6 (7.4)	0.381
mMRC score(≥2)	48 (60.0)	45 (55.6)	0.341
CAT score	19.55 ± 7.26	19.15 ± 7.12	0.723
24-h sputum volume, mL	28.84 ± 40.94	29.74 ± 41.35	0.890
Etiology of bronchiectasis			0.179
Postinfectious	38 (47.5)	30 (37.0)	
Idiopathic	42 (52.5)	51 (63.0)	
HRCT grade, n (%)			0.194
1	28 (35.0)	27 (33.3)	
2	41 (51.2)	34 (42.0)	
3	11 (13.8)	20 (24.7)	
Number of lesion lobes, n (%)			0.822
1 lobe	15 (18.8)	18 (22.2)	
2–3 lobes	47 (58.8)	44 (54.3)	
4–6 lobes	18 (22.5)	19 (23.5)	
Cystiform bronchiectasis, n (%)	33 (41.2)	44 (54.3)	0.097
*Pseudomonas aeruginosa* positive, n (%)	20 (25.0)	27 (33.3)	0.245
Medications, n (%)			
Inhaled corticosteroids and long-acting β-agonist	45 (56.2)	56 (69.1)	0.091
Inhaled short-acting β-agonist	20 (25.0)	15 (18.5)	0.391
Inhaled anticholinergics	22 (27.5)	24 (29.6)	0.765
Inhaled corticosteroids	17 (21.2)	11 (13.6)	0.199
Prednisone	2 (2.5)	3 (3.8)	1.000
Theophylline	6 (7.5)	4 (5.0)	0.746
Pulmonary function			
FVC, L	2.42 ± 0.94	2.32 ± 0.74	0.483
FEV_1_, L	1.56 ± 0.81	1.62 ± 0.73	0.629
Predicted FEV_1_, %	63.63 ± 26.28	60.23 ± 27.32	0.451
FEV_1_/FVC, %	64.39 ± 14.63	67.44 ± 16.49	0.226
Inspiratory capacity, L	1.77 ± 0.70	1.88 ± 0.81	0.368
ESR, mm/h	25.39 ± 19.86	27.53 ± 24.07	0.540
CRP, mg/dL	16.99 ± 21.26	13.37 ± 17.12	0.246
Number of exacerbations in the last year	2 (2–3)	2 (2–3)	0.713
Bronchiectasis Severity Index	8.00 ± 4.27	8.43 ± 4.68	0.548

Data are n (%) or mean ± SD or median (IQR). Abbreviations: *mMRC* modified Medical Research Council, *CAT* chronic obstructive pulmonary disease assessment test, *HRCT* high resolution computed tomography, *FVC* forced vital capacity, *FEV*_*1*_ forced expiratory volume in 1 s, *ESR* erythrocyte sedimentation rate, *CRP* C-reactive protein

### Efficacy

#### Primary end-point

**Fig. 2:**
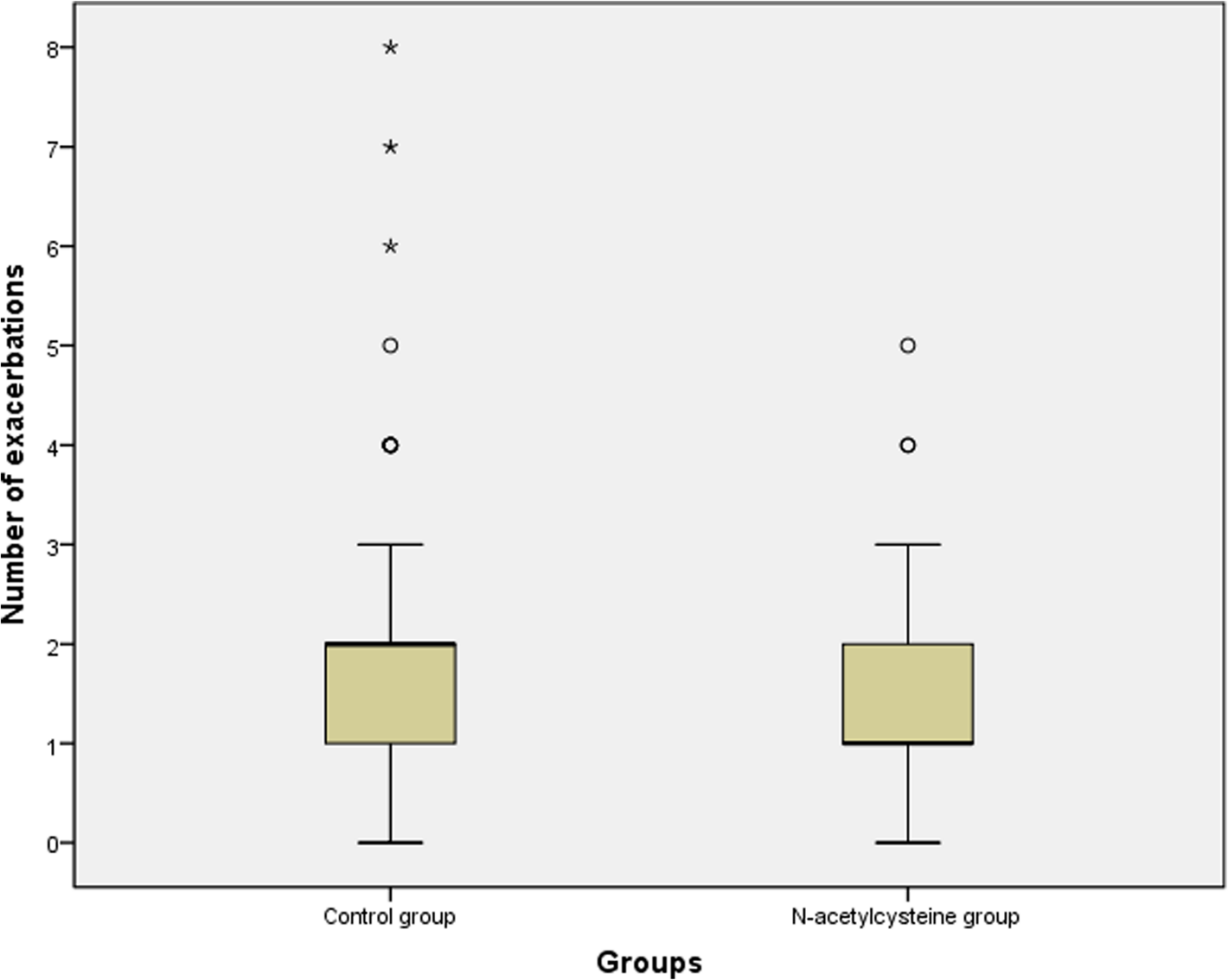
Number of exacerbations for the control and N-acetylcysteine groups

Eighty-one patients in the N-acetylcysteine group and 80 patients in the control group were included in the primary outcome analysis. The numbers of cumulative exacerbations for the two groups at the 12-month follow-up were 106 (the N-acetylcysteine group) and 158 (the control group), respectively. The incidence of exacerbations in the N-acetylcysteine group was significantly lower than that in the control group (1.31 vs. 1.98 exacerbations per patient-year; risk ratio, 0.41; 95% CI, 0.17–0.66; *P* = 0.0011). The median number of exacerbations in the N-acetylcysteine group was 1 (IQR, 0.5–2), compared with 2 (IQR, 1–2) in the control group (U = − 2.95, *P* = 0.003 by the Mann-Whitney U test) (Fig. 2).

#### Secondary end-points

**Fig. 3:**
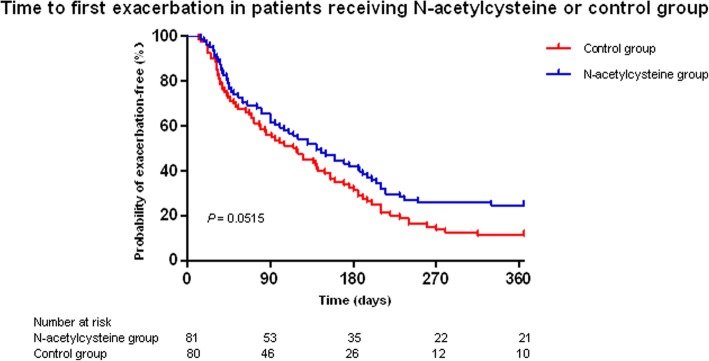
Time to first exacerbation in patients receiving N-acetylcysteine or control group

**Fig. 4:**
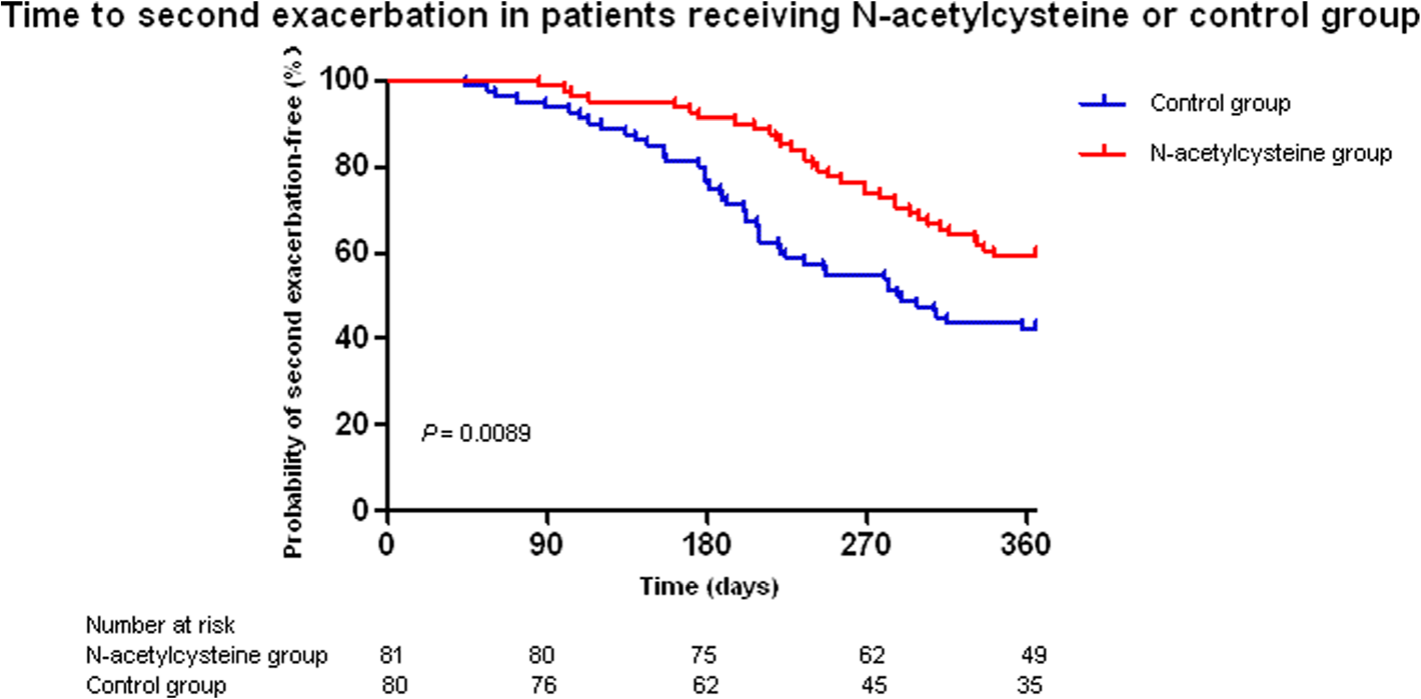
Time to second exacerbation in patients receiving N-acetylcysteine or control group

A total of 24.7% of the N-acetylcysteine group patients and 11.3% of the control group patients remained exacerbation-free throughout the 12-month follow-up period (*χ*^*2*^ = 4.924, *P* = 0.026). The time to the first exacerbation did not differ between the N-acetylcysteine group and the control group (*χ*^*2*^ = 3.795, *P* = 0.0515, Fig. 3). However, the time to the second exacerbation was longer in the N-acetylcysteine group than in the control group (*χ*^*2*^ = 6.849, *P* = 0.0089, Fig. 4). Compared with the control group, the volume of 24-h sputum in the N-acetylcysteine group was significantly reduced (*t* = − 3.091, *P* = 0.002) (Table 2). In addition, the N-acetylcysteine group showed a significant improvement in the quality of life (*t* = − 2.57, *P* = 0.011) (Table 2). However, there was no significant difference between the two groups in terms of the change of the number of patients with a positive sputum culture for *P. aeruginosa* from the baseline. The changes of inflammatory markers, including the serum CRP levels and ESR, during the study period were not significantly different between the two groups. Similarly, there were no significant differences in lung function between the two groups.

**Table 2:** Change from baseline parameters after the 12-month follow-up for the N-acetylcysteine and control groups

	Control group	N-acetylcysteine group	*P* value
CAT score	−1.44 ± 6.19	−3.79 ± 5.40	0.011
24-h sputum volume, mL	−6.46 ± 22.73	−18.28 ± 25.69	0.002
ESR	−0.36 ± 8.74	−4.21 ± 10.57	0.115
CRP	−1.68 ± 9.62	−2.83 ± 6.68	0.089
*Pseudomonas aeruginosa* positive, n (%)	−5 (25.0)	−8 (29.6)	0.726
Pulmonary function			
FVC, L	0.03 ± 0.22	0.01 ± 0.46	0.991
FEV_1_, L	0.03 ± 0.16	−0.10 ± 0.37	0.210
Predicted FEV_1_, %	0.13 ± 7.78	1.16 ± 16.50	0.445
FEV_1_/FVC, %	−0.29 ± 4.29	0.53 ± 7.45	0.394
Inspiratory capacity, L	0.01 ± 0.22	0.06 ± 0.24	0.098

Data are n (%) or mean ± SD. Abbreviations: *CAT* chronic obstructive pulmonary disease assessment test, *FVC* forced vital capacity, *FEV*_*1*_ forced expiratory volume in 1 s, *ESR* erythrocyte sedimentation rate, *CRP* C-reactive protein

#### Adverse events

The most common adverse events observed in this study are shown in Table 3. After treatment with N-acetylcysteine, three patients experienced mild elevation of liver enzymes and two patients had a rash, which were relieved by application of hepatoprotective or antiallergic agents. Serious adverse events were not observed during the study period. In addition, five patients in the N-acetylcysteine group complained of body odor during the treatment. It is likely that the odor of hydrogen sulfide was released after the decomposition of N-acetylcysteine in the gastrointestinal tract. The appearance of symptoms such as nausea, vomiting, and epigastric discomfort may also be related to this decomposition.

**Table 3:** Adverse events

Adverse event	Control group (*N* = 80)	N-acetylcysteine group (*N* = 81)
Epigastric discomfort	6 (7.5%)	8 (9.9%)
Abdominal pain	2 (2.5%)	2 (2.5%)
Vomit	4 (5%)	1 (1.3%)
Body odor	0	5 (6.2%)
Anorexia or nausea	8 (10%)	7 (8.6%)
Diarrhea	3 (3.8%)	1 (1.2%)
Rash	1 (1.3%)	2 (2.5%)
Dyspnea	5 (6.3%)	2 (2.5%)
Hepatic dysfunction	2 (2.5%)	3 (3.7%)

## Discussion

The results of this study showed that oral N-acetylcysteine treatment for 1 year in patients with bronchiectasis was associated with a reduction in the incidence of exacerbations compared with the control group in Shandong Province, China. In addition to the protective effect of N-acetylcysteine against exacerbations, this treatment at a dose of 600 mg twice daily had the advantages of reducing the volume of sputum and improving the quality of life of the bronchiectasis patients.

It is reported that the average frequency of exacerbations for bronchiectasis ranges from 1.5 to 6.5 per year [5]. Frequent exacerbations have a negative impact on long-term clinical outcomes for bronchiectasis patients, including lung function deterioration, increased risk of hospitalization, and increased medical costs [26, 27]. Therefore, there is an urgent need for clinical research dedicated to reducing the frequency of exacerbations. More recently, numerous studies have focused on the long-term use of macrolides for the treatment of bronchiectasis. Several major trials, including EMBRACE, BLESS, and BAT, consistently showed the efficiency of macrolides (azithromycin or erythromycin) in reducing the frequency of exacerbations in patients with bronchiectasis [28–30]. However, the long-term use of macrolides increased the risk of bacterial resistance, with a high incidence of adverse events. More importantly, the rate of macrolide resistance and the incidence of gastrointestinal adverse effects in patients treated with azithromycin were 88 and 40%, respectively [30]. Therefore, the safety profile of a treatment strategy should also be considered in the management of bronchiectasis.

Impairment of the mucociliary system in bronchiectasis patients is responsible for the excessive secretion of mucus and long-term retention of the sputum. Recently, increasing numbers of studies have focused on mucoactive agents. European Respiratory Society guidelines recommend offering long-term mucoactive treatment (≥3 months) in adult patients with bronchiectasis [20]. However, RhDNase is not recommended by either the European Respiratory Society guidelines or the British Thoracic Society guidelines because there is evidence that rhDNase use is associated with worsening lung function in bronchiectasis [5, 20]. The quality of life was found to be improved by the use of mannitol or hypertonic saline in a subgroup of patients with bronchiectasis [9, 31]. Furthermore, Bilton et al. reported that the time to first exacerbation was prolonged in patients receiving mannitol as compared with the controls [32]. However, few clinical research studies have reported the efficacy of mucolytic agents on the prevention of exacerbations in bronchiectasis patients. One reason might be the short-term therapy employed in previous trials, and another reason is that a certain subgroup of bronchiectasis patients might benefit more from mucoactive drugs. To the best of our knowledge, studies on the long-term use of N-acetylcysteine for the treatment of bronchiectasis have not been reported. In contrast to previous studies focusing on the use of other mucolytic agents in the treatment of bronchiectasis, the present study investigated N-acetylcysteine because it is not only an effective mucolytic agent but also has anti-inflammatory and antioxidant properties.

The role of N-acetylcysteine has been widely investigated in many studies involving patients with COPD. Both the PANTHEON and HIACE trials found that 1200 mg of N-acetylcysteine, daily, decreased the exacerbation frequency in Chinese patients with COPD [15, 33]. Consistent with the COPD studies, our study found that the long-term use of oral N-acetylcysteine (600 mg, twice daily, 12 months) prevented exacerbations in patients with bronchiectasis in Shandong Province, China, accompanied by a decrease of the daily sputum volume and improvement in the quality of life. We supposed that the underlying mechanism of N-acetylcysteine in preventing exacerbations might be mediated via the reduced volume of sputum so that the “vicious cycle” was broken down. Furthermore, the anti-inflammatory and antioxidant properties of N-acetylcysteine were also responsible for the results. In addition, evidence from in vitro studies indicates that N-acetylcysteine has the ability to interfere with biofilm formation and disrupt biofilms so that the survivability of bacteria in the respiratory tract can be inhibited [34].

Although our results indicated that the time to the first exacerbation did not differ between the N-acetylcysteine group and the control group, significantly more patients remained exacerbation-free in the N-acetylcysteine group than in the control group. Furthermore, the time to the second exacerbation was longer in the N-acetylcysteine group than in the control group. Our findings are consistent with those of the long-term PANTHEON study [15]. The positive cultures for *P. aeruginosa* were not different between the two groups. To investigate the difference of positive cultures, patients need to be stratified according to the results of the baseline bacterial culture. Patients with chronic colonization of *P. aeruginosa* are more likely to benefit from the treatment. In addition, no improvement in pulmonary function was observed after N-acetylcysteine treatment for 1 year, which is consistent with the results of the PANTHEON and HIACE trials [15, 33]. Probably, reducing the frequency of exacerbations and improving the quality of life of the patients have nothing to do with the bronchodilation effect.

Similar to other clinical studies regarding the long-term use of N-acetylcysteine, our study found that long-term N-acetylcysteine therapy was safe. A low incidence of adverse reactions was reported in patients with bronchiectasis, indicating that N-acetylcysteine was well tolerated. In addition, it has been reported that N-acetylcysteine-related adverse drug reactions are mainly associated with anaphylactoid reactions to N-acetylcysteine [35]. Anaphylactoid reactions are nonimmunogenic and do not require prior sensitization. The precise mechanisms of these reactions remain unclear, although non-mast cell sources of histamine are likely to play a role [36]. Body odor and gastrointestinal adverse reactions were found in our study, but they did not affect the overall efficacy of N-acetylcysteine. Of note, the use of N-acetylcysteine can avoid the problems of gastrointestinal adverse reactions and bacterial drug resistance caused by long-term azithromycin therapy [30].

To the best of our knowledge, this BENE study was the first trial to explore the efficacy and safety of long-term use of N-acetylcysteine for the treatment of bronchiectasis in China. However, this study had several limitations. First, the subjects were enrolled from the same province in China, reflecting a rather homogenous population of bronchiectasis patients. There is some geographical variation in etiology, epidemiology, and microbiology of bronchiectasis [37]. Besides, inconsistent diagnostic criteria were used to study the etiology of bronchiectasis. As a result, the proportion of idiopathic bronchiectasis patients in the Chinese population [18, 38] seems to be higher than that in the Spanish registry, which reported that only 24% were idiopathic [39]. The prognosis and management of bronchiectasis might differ between various geographic regions. Therefore, the effect of N-acetylcysteine on a Chinese population might differ from that in other populations. Along with the small sample size, these factors may influence the results. Hence, larger trials of heterogenous populations of bronchiectasis patients are needed to validate our results. Second, this study did not compare N-acetylcysteine to a placebo. Thus, there is a concern for bias. For example, N-acetylcysteine might also have been used in the control group. In addition, both researchers and patients were not blinded to the interventions, which may introduce the placebo effect. Due to these methodological flaws, the recommendation of long-term use of N-acetylcysteine in bronchiectasis cannot be made until the current results are validated in larger international multicenter trials. Third, this study did not analyze the therapeutic effects of N-acetylcysteine separately for “dry bronchiectasis,” “daily sputum,” or other subtypes of bronchiectasis. Since sputum production and chronic infection were less frequent in the “dry bronchiectasis” cluster, it is unclear whether the “dry bronchiectasis” group would benefit from the long-term use of N-acetylcysteine. Subgroup analysis of bronchiectasis according to microbiology and daily sputum suggests that different clinical phenotypes of bronchiectasis might exhibit diverse treatment outcomes in the same trial [6]. Different clinical phenotypes may require different treatments. Further studies are needed to explore how they are affected by treatment. Finally, nebulized N-acetylcysteine might be an effective way to deliver the drug, in contrast to oral administration, which involves first-pass liver metabolism and may result in a decreased efficacy or an increased toxicity. Further studies are needed to explore the efficacy and safety of nebulized N-acetylcysteine for bronchiectasis patients.

In conclusion, the long-term use of N-acetylcysteine is able to reduce the risk of exacerbations for bronchiectasis patients in Shandong Province, China. This trial may lead to larger international multicenter research in the field.

## Abbreviations

BSI: Bronchiectasis Severity Index
CAT: COPD assessment test
CIs: Confidence intervals
COPD: Chronic obstructive pulmonary disease
CRP: C-reactive protein
ESR: Erythrocyte sedimentation rate
FEV_1_: Forced expiratory volume in 1 s
FVC: Forced vital capacity
HRCT: High-resolution computed tomography scans
IQR: Interquartile range
mMRC: Modified Medical Research Council
